# Polymorphic Transitions in Cerium-Substituted Zirconolite (CaZrTi_2_O_7_)

**DOI:** 10.1038/s41598-017-06407-5

**Published:** 2017-07-19

**Authors:** Braeden M. Clark, S. K. Sundaram, Scott T. Misture

**Affiliations:** Kazuo Inamori School of Engineering, Alfred University, Alfred, NY 14802 USA

## Abstract

Compounds with the formulae CaZr_1−x_Ce_x_Ti_2_O_7_ with x = 0.1–0.5 were synthesized by solid state reaction. Cerium was used as a surrogate for actinide elements. A transition from the 2M polymorph to the 4M polymorph (expanded unit cell due to cation ordering) in zirconolite was observed with increasing cerium content. The presence of both tri- and tetravalent Ce, contrary to formulation, was confirmed using X-ray absorption near edge spectroscopy, suggesting substitution on both Ca and Zr sites. Sintering was carried out via spark plasma sintering, during which the perovskite phase (Ca_0.4_Ce_0.4_TiO_3_) was stabilized due to the reducing conditions of this technique. Scanning electron microscopy and energy dispersive spectrometry revealed that the 2M polymorph was dilute in Ce content in comparison to the 4M-zirconolite. High temperature X-ray diffraction was used to detail the kinetics of perovskite to zirconolite transition. It was found that CaCeTi_2_O_7_ (cubic pyrochlore) formed as an intermediate phase during the transition. Our results show that a transition from 2M- to 4M-zirconolite occurs with increasing Ce content and can be controlled by adjusting the P_O2_ and the heat treatment temperature.

## Introduction

Zirconolites (CaZrTi_2_O_7_) are potential crystalline phase host materials for both tri- and tetravalent actinides, either as single phase waste forms or as part of a multiphase assemblage^1–4^. For example, Cm-doped CaZrTi_2_O_7_ shows swelling and amophization due to self-radiation damage^5^. The valence state of the substituting actinide ions will affect the site of substitution as well as the quantity of the ions (waste loading) that can be incorporated. Therefore this information is critical for the informed design of waste forms.

Cerium oxide is typically used as a surrogate material for plutonium oxide (and other actinide elements) in simulated nuclear waste forms^6^ due to the similar electronic structure, ionic size and multiple valence states of both Ce and Pu. Both Ce and Pu can form tetravalent oxides with a fluorite structure and a trivalent phosphate with a monazite structure when heated in air between temperatures of 1000–1400 °C^7^, demonstrating similar reduction potential of the two elements even in an oxidizing atmosphere; however, it should be noted that the propensity to reduce is higher for Ce. Cerium oxide has been deemed as a ‘suitable’ surrogate for plutonium oxide by researchers based on the similar sintering behavior and phase assemblages during reaction^8^. Others found Ce to be a good surrogate for pyrochlore materials when the synthesis of the material took place in an oxidizing atmosphere and acceptable for zirconia-based ceramics; however, the use of Ce as a surrogate in zircon-based ceramics was considered limited^9^. Despite conflicting reports on the applicability of Ce as a surrogate for Pu, it is still widely used in simulated waste forms.

The incorporation of Ce into zirconolite materials has been studied previously. Vance and coworkers^10^ synthesized zirconolite materials in a reducing atmosphere and found that Ce could be successfully substituted onto the calcium site as a trivalent ion in small quantities, Ca_0.8_Ce_0.2_ZrTi_1.8_Al_0.2_O_7_, when using aluminum as a compensating ion. When tetravalent Ce was targeted on the zirconium site (CaZr_0.8_Ce_0.2_Ti_2_O_7_), two zirconolites were reported to be formed, with one polymorph containing a majority of the Ce. No valence data was reported.

In 1997, Begg^11^ reported formation of two zirconolites when targeting tetravalent Ce on the zirconium site (CaZr_0.8_Ce_0.2_Ti_2_O_7_). The synthesis of these materials was performed in air as opposed to the reducing atmosphere used in Vance’s experiments. The new zirconolite phase that was formed belonged to the 4M (monoclinic) polymorph as opposed to the 2M polymorph.

**Figure 1:**
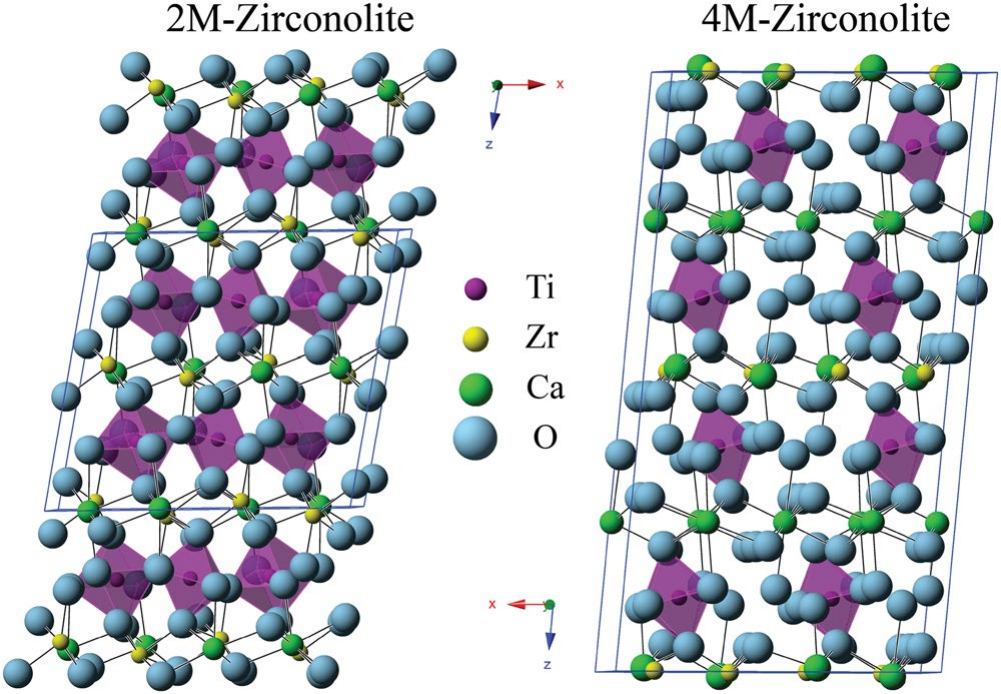
2M and 4M polymorphs of zirconolite, showing only the fully occupied Ti sites.

2M-ziconolite has been described by Rossel^12^ as having an anion deficient fluorite structure with layers of corner sharing TiO_6_ octahedra that are separated by layers containing ZrO_7_ and CaO_8_ polyhedra. This polymorph forms with compositions of CaZr_x_Ti_3−x_O_7_ with 0.8 < x < 1.37. The 4M-zirconolite is described by Coelho^13^ as having an enlarged unit cell (with c-axis of ~22 Å compared to ~11 Å of 2M) due to the cation occupancy. There are two full occupancy Ti sites in the 2M structure, opposed to only one full occupancy Ti site in the 4M structure, requiring a doubling of the c axis to accommodate. The 4M-zirconolite is formed when cations (such as Nd used by Coelho^13^) substitutes on both the calcium and zirconium sites simultaneously with 0.5–0.8 formula units creating an alternate layering of the structure. The structures are compared in Fig. 1.

In addition to the previously mentioned studies, the incorporation of cerium into zirconolite and related pyrochlore structures has been investigated by many other research groups, both experimentally and theoretically^14–18^. The tendency of Ce to take on both tri- and tetravalent states leads to the formation of ancillary phases when targeting a single phase, such as perovskite and cerianite^19^ and sphene and perovskite^20^.

Spark plasma sintering (SPS) is of interest in the processing of nuclear waste forms due to the lower temperatures and shorter time scales required to obtain high density samples compared to traditional pressureless solid state sintering and hot isostatic pressing (HIPing). Simulated waste forms have been fabricated in minutes (~30 minutes for the entire process) to high theoretical densities^21, 22^. The powder samples are contained within a graphite die during processing; this, along with the shorter processing times and lower processing temperatures, will limit volatile loss of radionuclides. The environment during the SPS process is inherently reducing due to the use of graphite die and hardware^23^.

Atomistic modeling can aid in the understanding of the Ce incorporation into materials for nuclear waste applications. Pyrochlore materials have been studied using different modeling methods^24–26^. In general, an energy minimization technique is used with Buckingham potentials to obtain a relaxed structure. One program that utilizes this technique is the General Utility Lattice Program (GULP). The program takes the internal energy (which is made up of coloumbic, polarizability, dispersion, etc.) and writes it as a Taylor series, calculates the derivative and the second derivative, and finds the path towards the local minimum.

Defect calculations are then performed to find the defect energies of substitutions using the Mott-Littleton technique^27^. This technique utilizes two regions surrounding the defect to calculate the energy. The defect is located in the center, or midpoint between multiple defects. Regions are specified by radii, to include an appropriate amount of ions. Ions in Region I (~150–200 atoms) are strongly affected by the defect and are explicitly using energy minimization and force balance techniques. Ions in Region II (~1000 atoms) are weakly perturbed and the atom positions are approximated. A larger Region I allows for more complete relaxation around the defect. The defect energy is calculated by taking the difference in energy between perfect regions and the defective regions.

This papers reports the evolution of the 4M-zirconolite with increasing Ce content, subsequent consolidation via spark plasma sintering (SPS) and the phase evolution post-sintering, supplemented with Ce valence data and defect calculations using GULP. This work also captures the 2M-4M dynamics in zirconolite as a function of Ce substitution and processing.

## Experimental

### Material preparation

Solid state synthesis of CaZr_1−x_Ce_x_Ti_2_O_7_ materials was used for material preparation. High purity oxide precursors of CaO, ZrO_2_, TiO_2_ and CeO_2_ (Alfa Aesar, MA, USA) were used in the experiments. The formula CaZr_1−x_Ce_x_Ti_2_O_7_ was used with x = 0.1–0.5. The appropriate amount of precursor material was weighed and placed in a ZrO_2_ milling jar with 2 mm YSZ milling media (Across International, NJ, USA). The precursors were milled at 1,200 rpm using a mixture of ethanol and water as the milling medium in a VQ-N High Speed Ball Mill (Across International) for 20 min. The resulting slurry was separated from the media and dried on a hot plate. The dry powders were homogenized using a mortar and pestle and pressed into 20 mm diameter pellets using a hydraulic press and a steel die. The pellets were reacted in a furnace at 1350 °C for 72 h, crushed into powder, milled for 5 min using the same procedure, dried, and homogenized. Scanning electron microscopy (SEM) with energy dispersive spectroscopy (EDS) was performed on polished sections of the synthesized pellets.

### Phase content determination

Powdered materials were analyzed with X-ray diffraction (XRD) with a D8 Advance instrument (Bruker) over a range of 15–115°2θ with a step size of 0.03°2θ and a count time of 1 s using a Lynxeye position-sensitive detector. The pattern was evaluated with Rietveld analysis using the program TOPAS (Bruker). The XRD patterns were fitted to CaZrTi_2_O_7_ (2M-zirconolite), CaZrNdTi_2_O_7_ (4M-zirconolite), and Ca_0.4_La_0.4_TiO_3_ (perovskite) with PDF cards #04-002-4312, 04-009-5863, and 00-055-0841, respectively. The relative phase amounts of the materials were calculated from the Rietveld scale factors.

### Atomistic modeling (GULP)

The GULP program was used to explore the substitution mechanism of Ce into zirconolite and pyrochlore materials. The structure for 2M-zirconolite and other structures of the oxide precursors were first relaxed using Buckingham potentials for the element-oxygen interactions found in the literature^28^. The values of the lattice constants and density from the literature and after relaxation using GULP are listed in Table 1. The energy for defects that could potentially occur in the structure were calculated using GULP and the total energy of the defects were calculated according to the specific defect equations.

**Table 1:** Values of lattice constants and densities for zirconolite and pyrochlore materials found in the literature and after structural relaxation using GULP.

Property	Experimental^12^	Calculated
Lattice Volume (Å)	1014.06	1016.17 (2.1)
a (Å)	12.4458	12.93 (3.9)
b (Å)	7.2734	7.13 (−2.0)
c (Å)	11.3942	11.29 (−0.9)
β (°)	100.533	102.4 (1.9)
ρ (g/cm^3^)	4.4418	4.433 (−2.1)

*Percent variation in parentheses.

### Spark plasma sintering (SPS)

Consolidation of Ce-zirconolites was performed using SPS on an FCT HP D 25 (FCT Systeme GmbH, Germany) furnace with graphite dies and punches. Powdered samples were loaded into graphite die lined with graphite foil with a diameter of 18.85 mm. Samples were sintered using a heating and cooling rate of 100 °C/min to a maximum temperature of 1100 °C (as read by a pyrometer facing the outer die wall), with a hold time at maximum temperature for 5 min and an applied pressure of 98 MPa during the run (applied before heating). Graphite foil remained adhered to the resulting sintered pellet and was removed by grinding with SiC grit paper prior to XRD measurements.

### High temperature X-ray diffraction (HTXRD)

Phase conversion after SPS was studied using HTXRD using a Siemens D5000 diffractometer equipped with a custom high temperature stage^29^. A SPS sample of CaZr_0.6_Ce_0.4_Ti_2_O_7_ composition was powdered and mounted on an alumina sample holder by mixing with isopropanol. Table 2 shows two time-temperature profiles used to investigate the phase conversion.

**Table 2:** HTXRD schedules used to study phase conversion of Ce-zirconolite post-SPS.

Schedule	Ramp Rate (°C/min)	Max Temperature (°C)	Hold Time (h)	Scan Rate at T (scan/h)
1	30	1300	1	3
2	30	1350	6	2

### X-ray absorption near edge spectroscopy (XANES)

The valence state of Ce in the zirconolite samples was determined with XANES performed at the Cornell High Energy Synchrotron Source (CHESS). The Ce L_III_-edge was measured in fluorescence at room temperature. The scans were collected in 0.25 eV steps from 5685–5785 eV. The amounts of tri- and tetravalent Ce present was determined by a linear relationship between the edge energies of CeO_2_ (99.99%, Alfa Aesar) and CePO_4_ (99%, Alfa Aesar) powders used as standards.

## Results and discussion

**Figure 2:**
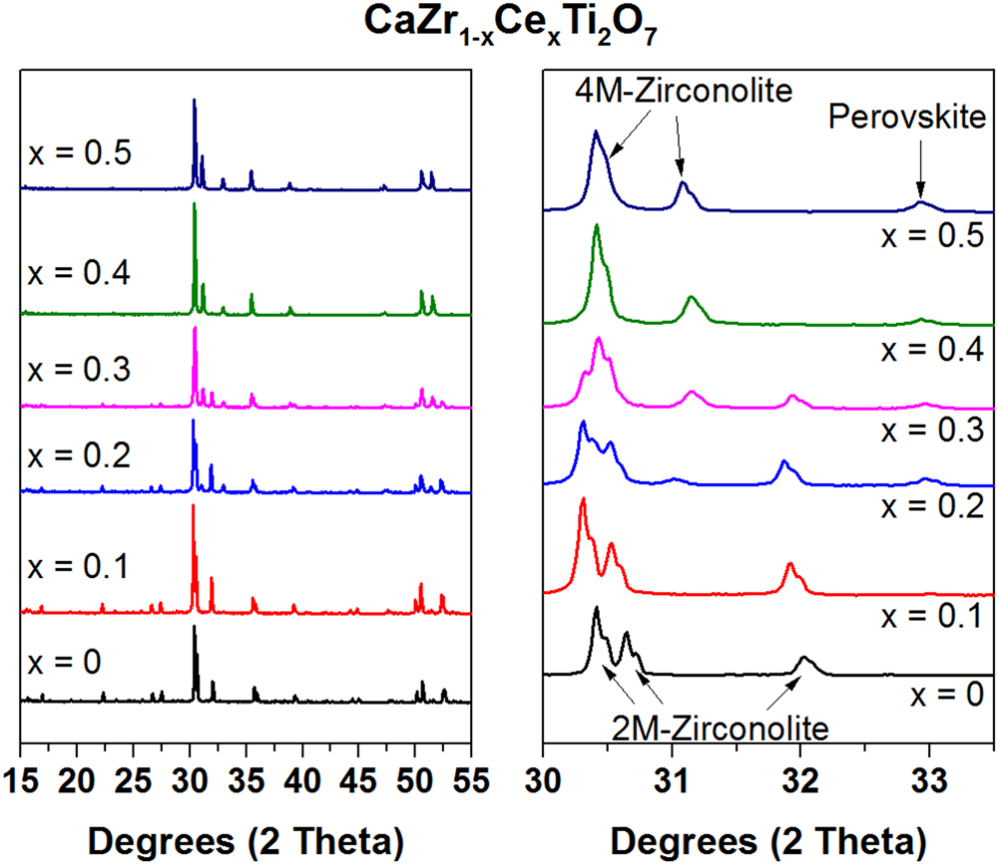
XRD patterns of Ce-zirconolites.

Figure 2 shows the XRD patterns of zirconolite samples with Ce substitution from x = 0–0.5, targeting the zirconium site. The amount of 4M-zirconolite (compared to 2M-zirconolite) is seen to increase with greater Ce content. Rietveld analysis confirms this trend and the results are listed in Table 3. By x = 0.5, the zirconolite is present in the 4M polymorph. A small amount of perovskite (Ca_0.4_Ce_0.4_TiO_3_) is also seen in all of the samples. This is most likely due to the substitution of Ce on the calcium sites, making excess calcium available to react contrary to the formulation.

**Table 3:** Percentages of phases present in zirconolite powder samples synthesized via solid-state sintering determined by Rietveld analysis.

Sample	2M-Zirconolite	4M-Zirconolite	Perovskite (Ca_0.4_Ce_0.4_TiO_3_)
CaZr_0.9_Ce_0.1_Ti_2_O_7_	99.0(1) wt%	0 wt%	0.9(1) wt%
CaZr_0.8_Ce_0.2_Ti_2_O_7_	74.5(3) wt%	19.3(3) wt%	6.2(1) wt%
CaZr_0.7_Ce_0.3_Ti_2_O_7_	45.6(3) wt%	49.0(3) wt%	5.4(1) wt%
CaZr_0.6_Ce_0.4_Ti_2_O_7_	3.6(3) wt%	89.4(3) wt%	6.9(2) wt%
CaZr_0.5_Ce_0.5_Ti_2_O_7_	0 wt%	95.6(4) wt%	4.3(3) wt%

**Figure 3:**
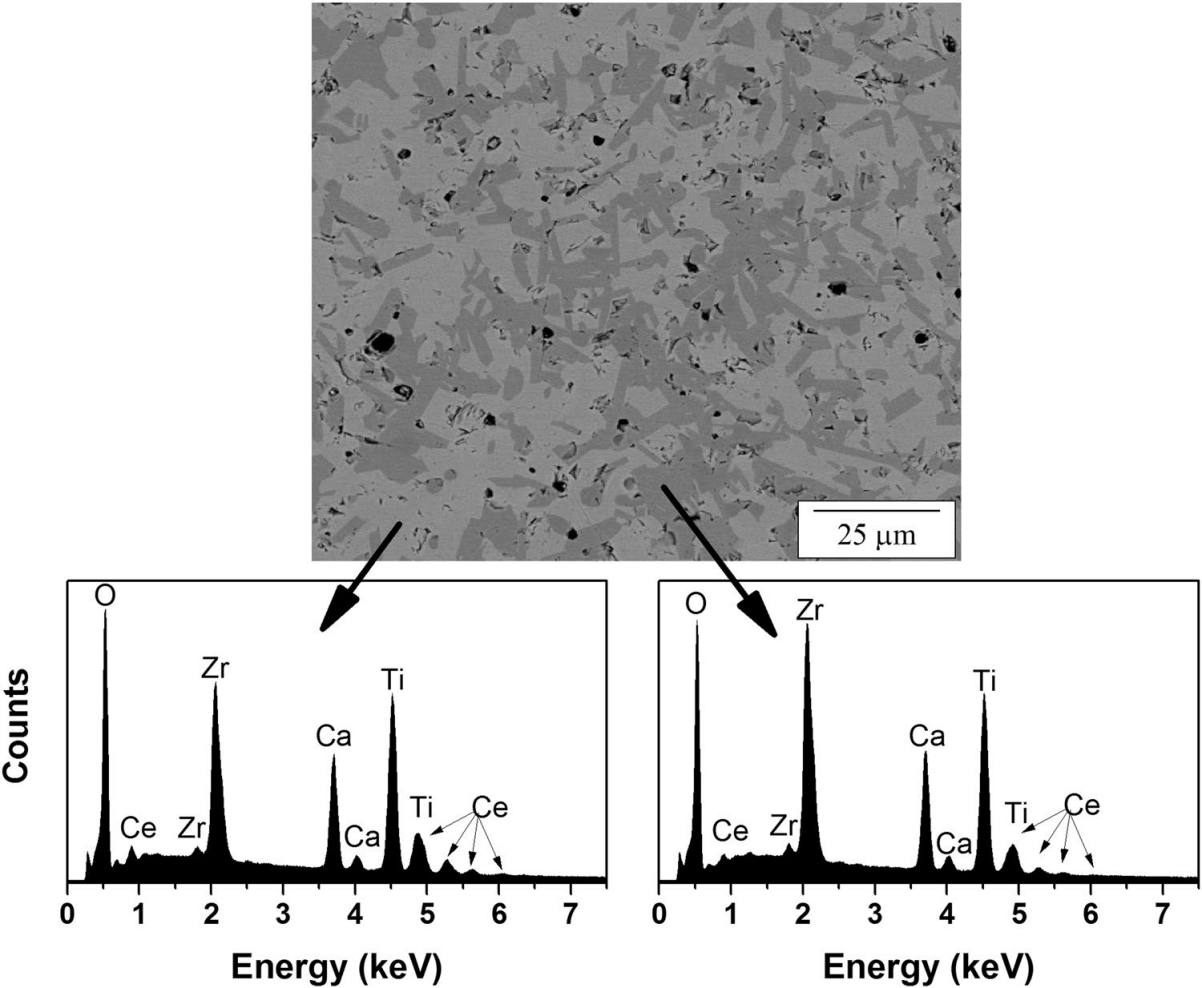
Representative BSE image of synthesized CaZr_0.7_Ce_0.3_Ti_2_O_7_ and corresponding EDS spectra from the two phases seen.

A representative SEM image of synthesized CaZr_0.7_Ce_0.3_Ti_2_O_7_ taken in backscatter electron (BSE) mode is displayed in Fig. 3. Two phases can clearly be distinguished and are evenly distributed, which is in agreement with XRD of roughly equal amounts of 2M- and 4M-zirconolite. The contrast in backscattered images suggests that the one polymorph contains a greater amount of Ce than the other. This is confirmed with EDS, with roughly twice as much Ce content in the bright phase. The bright phase belongs to 4M-zirconolite because as the amount of substituted Ce increases, the amount of 4M-zirconolite also increases. Therefore, the darker phase belongs to 2M-zirconolite. The EDS also shows that the dark phase (2M-zirconolite) is richer in Zr, indicating that greater partitioning of Ce to the Zr site in 4M-zirconolite occurs.

**Figure 4:**
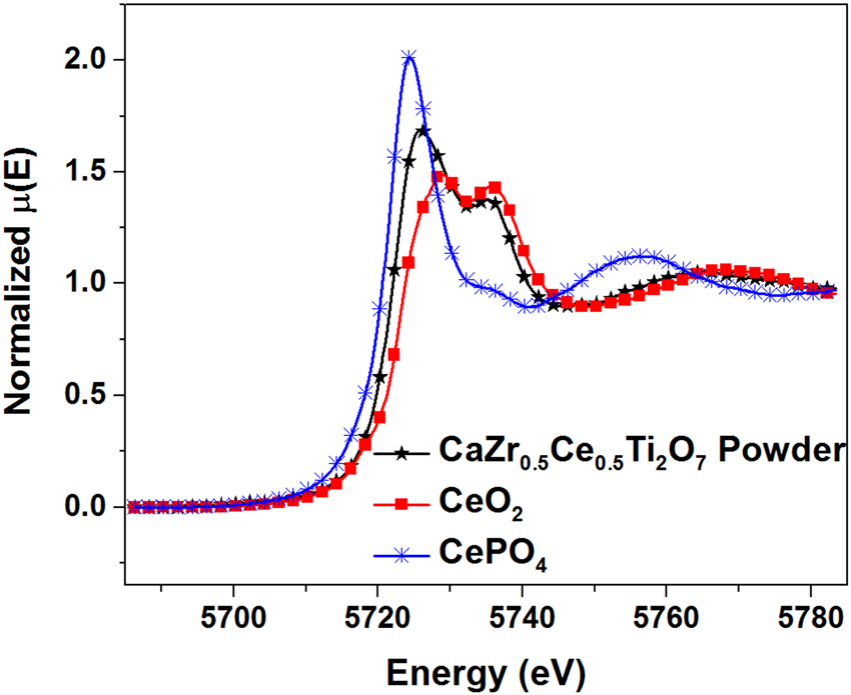
XANES results from standard materials and synthesized Ce-zirconolite powder.

XANES was performed on powdered samples of CaZr_0.5_Ce_0.5_Ti_2_O_7_ to detail the Ce valence state in these Ce zirconolites. The valence state of the Ce did not vary with composition, so only results from the x = 0.5 composition are shown in this paper. Figure 4 displays the XANES results from the standards, CeO_2_ and CePO_4_, and the synthesized powder. From the relationship between the edge energies, the synthesized powders contain about a 50/50 mix of tri- and tetravalent Ce. The presence of both tri- and tetravalent Ce demonstrates the ability of Ce to reduce at high temperatures in oxidizing atmospheres^11^. Trivalent Ce can co-substitute on both the Ca and Zr sites (as is seen in the 2M-zirconolite), and the tetravalent Ce can substitute for Zr (as in the 4M-zirconolite) without a need for a compensating ion. In zirconolite, this is often achieved by substituting Al for Ti^11, 30^.

Six possible substitution mechanisms for Ce into zirconolite listed in Table 4 were investigated with defect calculations using GULP. The heat of solution was calculated by calculating the lattice energies of the structures and the energy of the defects in the equation. The lower the heat of solution, the more likely the substitution is to occur.

**Table 4:** Possible substitution mechanisms for Ce incorporation into 2M-zirconolite.

Substitution Mechanism	Heat of Solution (eV)
2CeO_2_ → 2Ce_Ca_ + 2CaO + V_Zr_ + ZrO_2_	4.66
CeO_2_ → Ce_Zr_ + ZrO_2_	0.01
3CeO_2_ → 3Ce_Ca_ + 6CaO + 3V_Ca_	8.35
CeO_2_ → Ce_Ca_ + CaO + O_i_	6.94
2CeO_2_ → 2Ce_Ca_ + 2CaO + V_Ti_ + TiO_2_	5.99
Ce_2_O_3_ → Ce_Ca_ + Ce_Zr_ + CaO + ZrO_2_	3.90

These defect calculations show that the most likely substitutions to occur are for tetravalent Ce replacing Zr and trivalent Ce replacing one Ca and Zr simultaneously. This result supports our claim that greater Ce partitions to the Zr site, as there is both tri- and tetravalent Ce present in our samples (Fig. 4).

**Figure 5:**
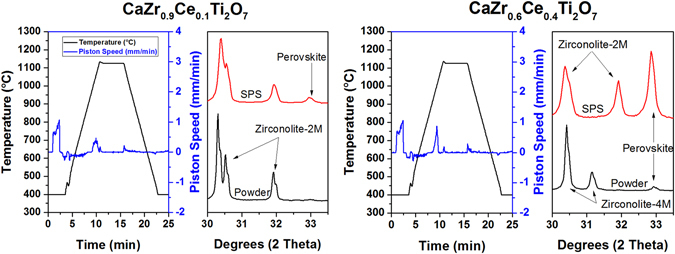
Temperature profile and piston speed during SPS of CaZr_0.9_Ce_0.1_Ti_2_O_7_ and CaZr_0.6_Ce_0.4_Ti_2_O_7_ including XRD comparison of the powder and resulting SPS pellet.

To consolidate the powder into monoliths, SPS was performed on the Ce-zirconolites. The sintering behavior of CaZr_0.9_Ce_0.1_Ti_2_O_7_ was very similar to single phase material, where rapid consolidation occurs immediately prior to reaching maximum temperature. XRD of the resulting pellet shows similar phases to starting powder with a small amount of perovskite phase (Fig. 5). The sintering peak of CaZr_0.6_Ce_0.4_Ti_2_O_7_ was ‘sharper’ than the single phase material, indicating more rapid sintering. XRD of the resulting pellet shows an increase of perovskite phase. The change in sintering behavior is attributed to the sintering of both zirconolite and perovskite. The sintering curve and XRD are also shown in Fig. 5. The destabilization of 4M-zirconolite in favor of 2M-zirconolite after sintering should also be noted.

**Figure 6:**
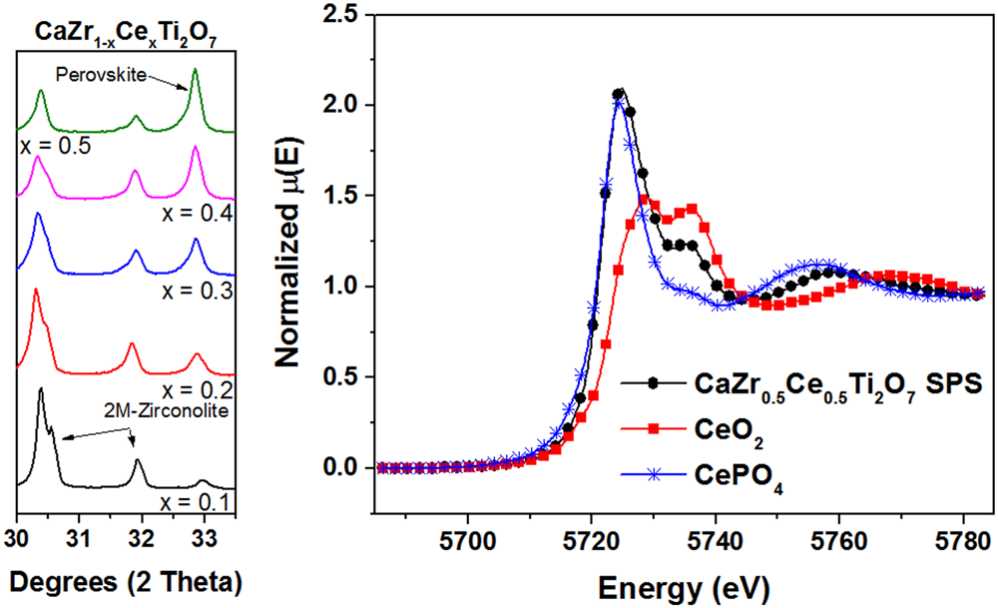
XRD comparison of SPS Ce-substituted zirconolites along with XANES results showing a majority of trivalent Ce.

This result demonstrates that the conversion to perovskite is a fast process, which occurs below the sintering temperature of zirconolite. SPS was performed on the range of Ce-substituted zirconolites. It was found that the amount of perovskite formed during SPS increases as the amount of Ce in the material increases. A comparison of the XRD patterns of the sintered Ce-substituted zirconolites is shown in Fig. 6. The stabilization of a perovskite phase from zirconolite in reducing conditions (as in the case of SPS) has been reported previously^10, 31^. XANES reveals that about 90% of the Ce is present in the trivalent state after SPS (Fig. 6). Zirconolite transformation into perovskite is due to the partial reduction of Ti^4+^ to Ti^3+^ in the zirconolite structure^31^. In order for charge compensation to occur in the zirconolite structure, Zr relocates to the Ca site, allowing excess Ca and Ti to react to form perovskite with the reduced Ce.

**Figure 7:**
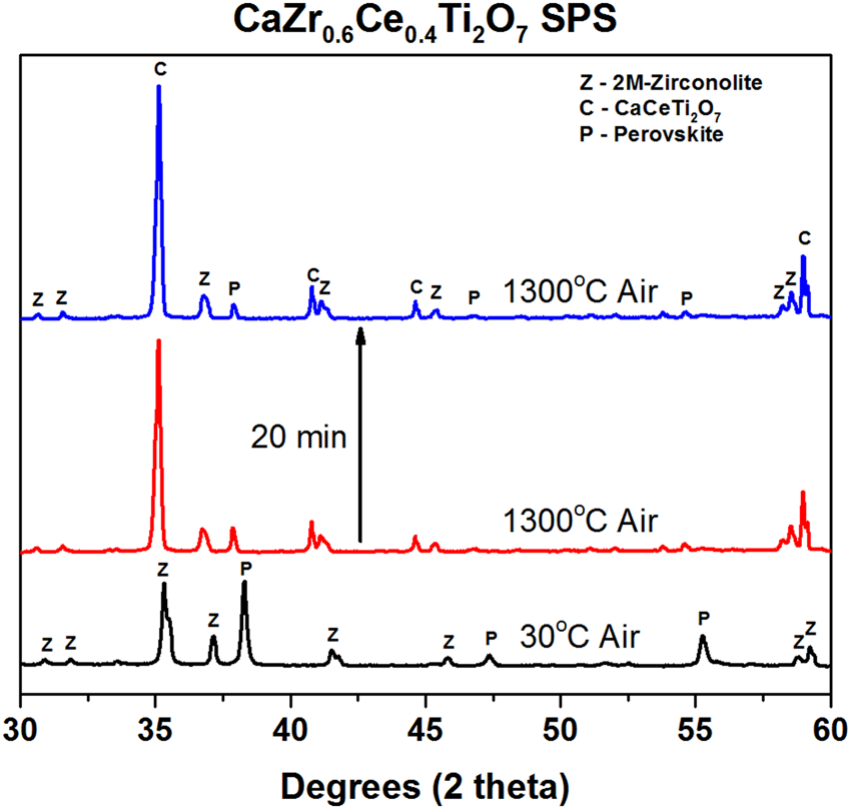
*In situ* XRD patterns of CaZr_0.6_Ce_0.4_Ti_2_O_7_ at 1300 °C showing the conversion of perovskite into CaCeTi_2_O_7_.

The formation of perovskite during SPS is undesirable due to the superior chemical durability of zirconolite^32^ compared to the perovskite phases. Thus, a heat treatment in air after SPS is required to convert the perovskite back into zirconolite. HTXRD was used to monitor the phase conversion during heat treatment. Schedule 1 (Table 2) reveals that CaCeTi_2_O_7_ forms as an intermediate phase at temperatures up to 1300 °C, as seen in the XRD patterns in Fig. 7. 4M-zirconolite does not form in the CaZr_0.6_Ce_0.4_Ti_2_O_7_ sample during Schedule 1. No significant changes to the patterns were seen in the subsequent measurement at 1300 °C.

**Figure 8:**
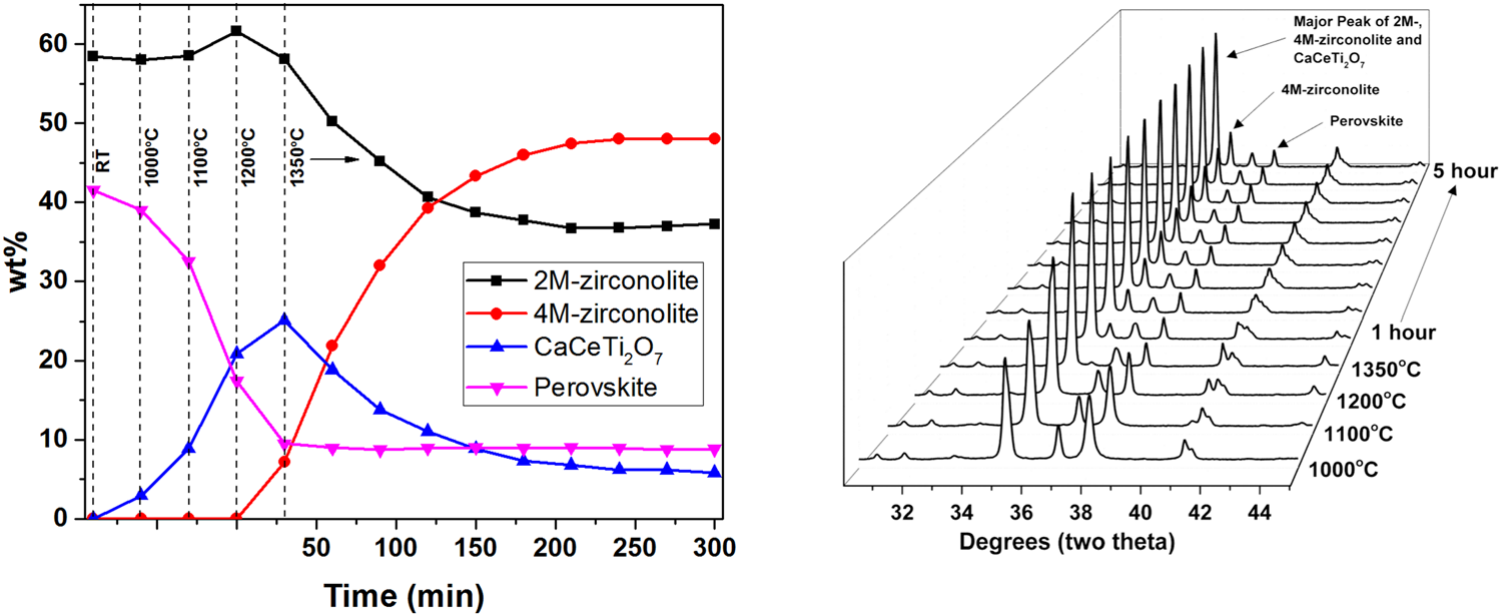
Phase amounts and corresponding XRD patterns during heating and isothermal hold at 1350 °C of CaZr_0.6_Ce_0.4_Ti_2_O_7_.

A second HTXRD schedule was performed to eliminate the CaCeTi_2_O_7_ that forms as an intermediate phase. At 1350 °C, CaCeTi_2_O_7_ and 2M-ziconolite are converted into 4M-zirconolite (Fig. 8). Rietveld analysis was performed on these patterns to determine the phase contents of the specimen during the 4M-zirconolite formation. Select diffraction patterns and the corresponding wt% determined by Rietveld analysis are shown in Fig. 8. It can be seen that perovskite reacts to form CaCeTi_2_O_7_ at temperatures below 1350 °C. During the 5 h of holding at 1350 °C, the perovskite content reaches a plateau at around 9 wt%. The CaCeTi_2_O_7_ and 2M-zirconolite react during the first 2 h of the hold at 1350 °C to form 4M-zirconolite which plateaus by around 3.5 h. The reaction did not go to completion (100% 4M -zirconolite) during the HTXRD experiment.

**Figure 9:**
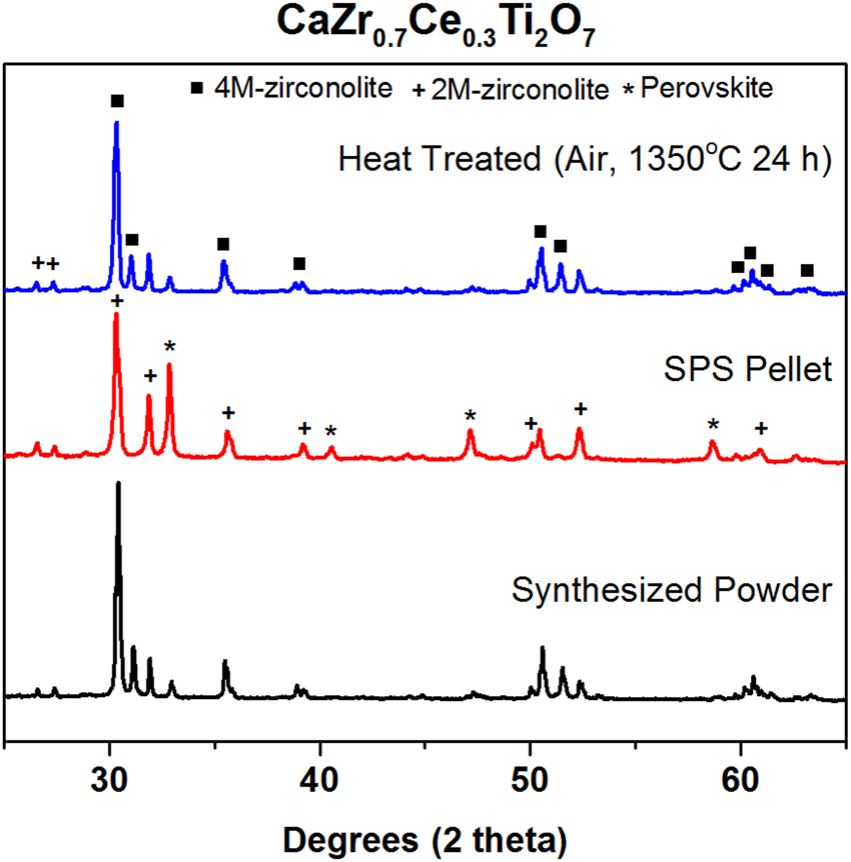
XRD patterns comparing the phase assemblage in CaZr_0.7_Ce_0.3_Ti_2_O_7_ under different processing conditions.

Conversion of the phases after SPS back to nearly the original phase composition was achieved by a furnace heat treatment at 1350 °C for 24 h. The longer heat treatment completes the conversion of CaCeTi_2_O_7_ into zirconolite (both 2M and 4M), which is desirable for the intended application. XRD patterns representing the phase changes are shown in Fig. 9.

## Conclusions

Ce incorporation into the zirconolite structure was confirmed using XRD and supporting data from SEM/EDS and XANES. A transition from the 2M polymorph to the 4M polymorph with increasing Ce substitution is evident. In 2M-zirconolite, trivalent Ce co-substitutes on both the Ca and Zr sites, while both trivalent (Ca and Zr sites) and tetravalent (Zr sites) Ce substitutes into 4M-zirconolite. Conversion of 4M-zirconolite to perovskite and 2M-zirconolite occurs during the SPS process due to the reducing environment. This is due to the reduction of Ce^4+^ into Ce^3+^, where the 2M-zirconolite is charge balanced by co-substitution of Ce^3+^ on the Ca and Zr sites and the perovskite forms due to the partial reduction of Ti^4+^ to Ti^3+^ leaving excess Ca an Ti to react with the trivalent Ce to form perovskite. HTXRD was used to study the transformation process of the perovskite into zirconolite in air. CaCeTi_2_O_7_ forms as an intermediate phase up until 1300 °C, and 4M-zirconolite begins to form at 1350 °C. The re-oxidation of Ce^3+^ and Ti^3+^ to their tetravalent states allows the original phase assemblage to be attained. The transformation to 4M-zirconolite is slow, but complete conversion to the original phase assemblage is achieved with a 24 h heat treatment in air.

### Data Availability

All data is available from the corresponding author upon reasonable request.
